# Barriers to healthcare for Australian autistic adults

**DOI:** 10.1177/13623613231168444

**Published:** 2023-05-10

**Authors:** Samuel RC Arnold, Georgia Bruce, Janelle Weise, Caroline J Mills, Julian N Trollor, Kristy Coxon

**Affiliations:** 1 UNSW Sydney, Australia; 2 Cooperative Research Centre for Living with Autism (Autism CRC), Australia; 3 Western Sydney University, Australia

**Keywords:** access, adult, autism, autistic, healthcare

## Abstract

Barriers to healthcare experienced by Australian autistic adults have not been previously explored. We conducted a cross-sectional investigation of barriers to healthcare and associated factors from a subtle realism perspective. Perceived barriers to healthcare were obtained from the Barriers to Healthcare Checklist Short-Form (BHC). A total of 263 autistic and 70 non-autistic individuals completed the BHC. On average, autistic adults reported more barriers to healthcare (4.58) than non-autistic adults (0.76). Gender diversity, higher levels of generalised anxiety, greater global disability and less satisfaction with social support contributed to the experience of barriers to healthcare in autistic participants in regression modelling. Australian autistic adults face substantial barriers to healthcare. Understanding these barriers provides an opportunity to develop approaches to improve access; such as co-designing a healthcare access roadmap for autistic adults, with co-designed policies and practices which advocate for the needs of autistic adults.

**Lay abstract:**

This study looked at how Australian autistic and non-autistic adults experience barriers to healthcare. We asked autistic and non-autistic adults to complete the Barriers to Healthcare Checklist Short-Form (BHC). We analysed data from 263 autistic adults and 70 non-autistic adults. We found that autistic adults experienced more barriers to healthcare than non-autistic adults. Gender diversity, feeling more anxious, having greater disability and feeling unsatisfied with social support contributed to barriers to healthcare in autistic participants. We recommend interventions such as developing and implementing a national action plan, similar to the National Roadmap for Improving the Health of People with Intellectual Disability (2021) to reduce barriers and address unmet healthcare needs of Australian autistic adults. We also recommend working with autistic adults to develop new policies and strategies, implementing environmental adaptations to health care facilities, and increasing Autism education opportunities for health professionals to address gaps in knowledge.

## Introduction

Autistic adults often have complex healthcare needs due to high rates of mental and physical co-occurring conditions, sensory processing disparities and differences in communication (Croen et al., 2015). They experience higher rates of mental and physical co-occurring illness than their non-autistic counterparts (Croen et al., 2015; Vohra et al., 2017). The presence of co-occurring conditions can lead to medical and financial burden in autistic adults, which can impede access to healthcare (Matson, 2016). Co-occurring physical illnesses are prevalent among autistic people with higher rates of conditions such as epilepsy, irritable bowel syndrome, hearing impairments and physical disabilities compared with the non-autistic population (Brondino et al., 2019). Co-occurring mental health conditions, such as anxiety and depression, are also high in the autistic population. In a US-based study by Croen et al. (2015), more than half the autistic adults in the sample were diagnosed with a psychiatric condition (*N* = 1507, mean age = 29), with 29% reportedly diagnosed with anxiety. These higher rates of co-occurring conditions may, in part, be a direct result of the challenges to accessing healthcare services that autistic people experience (Calleja et al., 2019).

The right to healthcare is universal and comprises seeking and receiving quality, non-discriminatory, affordable health services and information. This right extends beyond physical accessibility to encompass additional core constructs of availability, acceptability and quality (World Health Organization [WHO], 2008). Despite this universal right, health services are often designed to meet the needs of non-autistic people, with the recent Australian Royal Commission into Violence, Abuse, Neglect and Exploitation of People with Disability (2020) finding that ‘there has been, and continues to be, systemic neglect of people with cognitive disability in the Australian health system’ (p. 27). This has resulted in unmet health needs for the autistic population, as health professionals and services are underequipped to meet the complex and often unique needs of autistic people (Walsh, Lydon, O’Dowd et al., 2020).

Addressing these unmet health needs is critical as the prevalence of autism has been steadily increasing with autistic adults likely underrepresented in prevalence data (Evans, 2013). The unique needs of autistic people may mean they access healthcare differently compared with their non-autistic counterparts (Raymaker et al., 2017). Equitable health service access will be critical for the increasing number of autistic adults who will require it.

In Australia, the healthcare system has a mix of publicly and privately funded healthcare services. Public funding, known as Medicare, is complex and can lead to care co-ordination problems (i.e. difficulties coordinating multiple different health and allied health professionals to support the person in a timely and unified manner) with conflicting information given to beneficiaries (Dixit & Sambasivan, 2018). Although Medicare funding is provided by the federal government on the principle of ‘free universal healthcare’, not all costs are met, leaving healthcare consumers to pay out-of-pocket expenses, known as the Medicare gap. With finite fiscal and healthcare resources, access to public healthcare is often delayed by long treatment waiting lists – which can lead to poorer health outcomes for groups living with disability, such as autistic adults (Dixit & Sambasivan, 2018; Laba et al., 2015). Those who can afford private healthcare or private health insurance may access earlier treatment through private healthcare providers, while those unable to pay, wait. Out-of-pocket expenses for both public and private healthcare services create real financial barriers to accessing timely, appropriate and quality healthcare which disproportionally affect those with complex, concurrent or high-care medical needs, such as autistic adults (Callander et al., 2019). Navigating healthcare services in Australia is further complicated by each state and territory operating their healthcare systems differently.

Successful experiences with health services are largely facilitated by effective communication and clear understanding between the patient and practitioner (David & Gerhard, 2017). Verbal and non-verbal communication styles influence the way that an individual describes presenting problems to the practitioner (Nicolaidis et al., 2015). However, atypical verbal and non-verbal communication are defining characteristics of autism (Santhanam & Hewitt, 2021). If practitioners are unaware of how to adequately communicate with autistic adults seeking care, they may be unable to provide an appropriate standard of care.

Research on barriers to healthcare experienced by autistic adults’ highlights common challenges with lack of physician knowledge and formal training (Bruder et al., 2012; Mason et al., 2019). Healthcare providers report difficulty establishing rapport and implementing appropriate communication strategies with their autistic patients (Zerbo et al., 2015). As Mason et al. (2019) explain, social communication, practitioner assumptions and sensory processing differences present significant barriers for autistic adults when accessing health services. These common barriers experienced by autistic adults illustrate the need for further research into limitations and barriers to healthcare, especially within an Australian context, where research is particularly limited. Greater understanding of barriers to healthcare for autistic people across the lifespan is critical in ensuring that autistic people have successful experiences when engaging with healthcare facilities and services (Malik-Soni et al., 2021).

The aim of this study was to explore barriers to healthcare access for Australian autistic adults, compared with non-autistic Australians, and identify associations with barriers for autistic adults, to aid the development of targeted adjustments. Our research questions included the following: (1) what are the perceived barriers which limit access to healthcare by Australian autistic adults according to the Barriers to Healthcare Checklist Short-Form (BHC), (2) how do the perceived barriers to accessing healthcare as self-reported on the BHC differ between autistic and non-autistic Australian adults, and (3) what factors influence perceived barriers to healthcare experienced by Australian autistic adults?

## Methods

### Study design

The Australian Longitudinal Study of Autism in Adulthood (ALSAA) was a questionnaire-based longitudinal study completed at two timepoints and funded by the Cooperative Research Centre for Living with Autism (Autism CRC) (Arnold, Foley et al., 2019). The survey was available in three versions: self-report, informant-report and carer-report, and could be completed on paper or online. Only self-report responses at the second timepoint were used in this cross-sectional study. Ethical approval was obtained for this study from the Human Research Ethics Committee at UNSW Sydney, Australia (No. HC15001). Only researchers named on ethics approval had authorised access to raw data.

### Participants

Participants were autistic and non-autistic Australian adults aged 25 years or over who completed the ALSAA self-report survey at the second timepoint. The sister study, the Study of Australian School Leavers with Autism, which recruited participants aged 15–25 years, did not include the BHC, and data were unable to be pooled, hence younger adults were not included in this study. Through voluntary sampling, Australian adults were invited to partake in the survey through advertisement on the Autism CRC website, Universities, Technical and Further Education (TAFE) facilities (i.e. vocational education facilities), via autism specific organisations, aged care facilities and through health professionals. Snowball sampling (Sheu et al., 2009) was employed to increase recruitment numbers in timepoint two. There was no difference in recruitment efforts between autistic and non-autistic adults and no matching between samples occurred. Autistic and non-autistic people were eligible to enrol if they met the age criteria. Participants needed to currently reside in Australia and have sufficient English literacy skills to complete the survey, determined through a screening call or online expression of interest form. The autistic group was comprised of individuals with either a formal or self-diagnosis, who were included due to the underdiagnosis of autism historically (Lai & Baron-Cohen, 2015). Participants in the autistic group were removed if they did not meet the Autism Quotient-Short Form (AQ-Short) cut-off score, with no cut-off applied to the non-autistic group. In this study, we restricted participants to those who self-reported and were not missing any data in any of the predictor variables used in the regression model.

### Procedure

#### Data collection

ALSAA data used in the study was collected between 2017 and March 2021. Domains of data collected through the survey included demographics, autism characteristics, health and well-being, mental and emotional health, relationships and social networks, activities, participation and quality of life, service usage and caring. Survey completion was self-paced to minimise burden. The survey took approximately 2–3 h for participants to complete (Arnold, Foley et al., 2019).

#### Outcome measure

The BHC-short (Raymaker et al., 2017) provides insight into the experiences of autistic and non-autistic adults accessing healthcare. Derived from the long-form checklist, the short-form checklist includes 17 independent items exploring potential barriers to healthcare such as fear and anxiety about primary care visits, challenges communicating with providers or healthcare navigation. Items are scored by selecting ‘yes’ or ‘no’ to indicate presence or absence of a barrier. Scoring ranges from 0 to 17, with ‘yes’ scoring one and ‘no’ scoring 0 and greater scores indicating more barriers experienced when accessing healthcare. Good convergent validity was demonstrated during the tools development, with expected patterns of results between autistic adults, people with disability and a matched sample without disability. Collapsed short form items showed predominately high levels of correlation (range 0.2–0.8, *p* < 0.001) in the expected direction with their long form counterparts.

#### Demographics

Demographic information including age (in years), gender (male, female, other) and geographical location were included within the analysis to control for variables known to impact access to healthcare (Serban, 2019). Using the Australian Statistical Geography Standard, a postcode-based remoteness code was used to score participants remoteness into three classes: Major Cities of Australia; Inner Regional Australia and Outer Regional Australia; and Remote Australia and Very Remote Australia (Australian Bureau of Statistics, 2021).

### Autism characteristics

#### The AQ-Short

The AQ-Short (Hoekstra et al., 2010) is a 28-item self-report measure evaluating the severity of symptoms associated with autism – which may play an important role in accessing healthcare (Raymaker et al., 2017; Walsh, Lydon, Hehir, O’Connor, 2020; Walsh, Lydon, O’Dowd, O’Connor, 2020). Cronbach’s α values of AQ-Short total indicate acceptable-to-good internal consistency (α between 0.77 and 0.86). AQ-Short correlates very highly with the long-form version (*r* between 0.93 and 0.95) (Baron-Cohen et al., 2001). Scoring is based on four-point Likert-type scale, with options ranging from definitely agree to definitely disagree. The AQ-Short is scored out of 200, with a higher score indicating greater autism symptom severity. A score greater than 65 has a sensitivity of 0.97 and a specificity of 0.82 for the identification of autism (Hoekstra et al., 2010).

### Health and well-being

#### Patient Health Questionnaire-15 (PHQ-15)

The PHQ-15 assesses 15 somatic symptoms that account for over 90% of all physical complaints from outpatients (Han et al., 2011). Co-occurring conditions arising from such somatic symptoms can strongly influence healthcare utilisation and therefore access (Vohra et al., 2017). Cronbach’s α values of 0.87 indicate good internal consistency, and good test–retest reliability of 0.67 over a 2-week time period has been demonstrated (Han et al., 2011). Items are scored by selecting ‘not bothered at all’, ‘bothered a little’ or ‘bothered a lot’. The score of the PHQ-15 ranges from 0 to 30 with higher scores representing more severe levels of somatization.

#### Medical Outcomes Study Short-Form Health Survey Version 2 (SF-12)

The SF-12 Version 2 is a health-related quality-of-life survey, which measures the impact of health on everyday life. Autistic adults are more likely to experience co-occurring conditions which may impact health-related quality of life than their non-autistic counterparts (Croen et al., 2015). The SF-12 presented α = 0.83 and a good degree of reliability (Silveira et al., 2013). Items are a mix of ‘yes’ or ‘no’ and three- or five-point Likert-type scale questions. The SF-12 delivers several normed summary scores, with the two main scores being the Physical Component Scale (PCS) and the Mental Component Scale (MCS). For this study, we employed the PCS scored using New Zealand normative data (Frieling et al., 2013) as recent Australian normative data were unavailable. The SF-12 has been validated in a sample of autistic adults (Khanna et al., 2014).

### Mental and emotional health

#### Patient Health Questionnaire–9 (PHQ-9)

The PHQ-9 (Kroenke et al., 2001) is a nine-item, self-report measure, screening for the presence and severity of depressive symptoms. Due to the higher prevalence of depression among autistic people (Bergman et al., 2020), the PHQ-9 was included in this analysis to identify whether the severity, rather than just the presence of depression, impacts access to healthcare (Croen et al., 2015). Cameron et al. (2008) examined the effectiveness of the PHQ-9 measuring depression in primary care, α at baseline was 0.83 and 0.92 at end of treatment. PHQ-9 indicated convergent and discriminant validity and responsiveness to change (effect size 0.99). The PHQ-9 has also been validated in an autistic sample (Arnold, Uljarevic et al., 2019). Items are scored by selecting ‘not at all’, ‘several days’, ‘more than half of the days’ and ‘nearly every day’. PHQ-9 is scored out of 27, with higher scores indicating greater depression severity.

#### *DSM*-5 Dimensional Anxiety Scales: Generalised Anxiety Disorder–Adult (*DSM*-5 GAD-A)

The Diagnostic and Statistical Manual of Mental Disorders (5th ed.; *DSM*-5) GAD-A (Beesdo-Baum, Klotsche et al., 2012) is a nine-item self-report survey – which assesses anxiety symptomology in adults. The GAD-A has shown good internal consistency (α = 0.83), questionable test–retest reliability (*r* = 0.64 over 2 weeks) and convergent and divergent validity (Pierson et al., 2017). Items are scored by selecting ‘not at all’, ‘several days’, ‘more than half of the days’ and ‘nearly every day’. The measure is scored out of 21, with greater scores indicating greater levels of anxiety.

### Relationships and social networks

#### UCLA Loneliness Scale–8 (ULS-8)

The ULS-8 is an eight-item scale assessing loneliness. Research highlights the negative impact of loneliness on healthcare utilisation among adults (Gerst-Emerson & Jayawardhana, 2015). The ULS-8 has been validated, widely used and analysed including usage in autism research (Ee et al., 2019). It shows good internal reliability (α = 0.84) (Wu & Yao, 2008). Items are scored by selecting ‘never’, ‘rarely’, ‘sometimes’ or ‘always’. Total score ranges from 8 to 32 points, with higher scores suggesting a higher degree of loneliness.

#### Social Support Questionnaire (SSQ)

Our study used a six-item extract from the SSQ, which was determined to be applicable for an autistic cohort through consultation with the ALSAA Research Advisory Network (RAN). The SSQ is a measure of perceived social support and satisfaction with said support. Healthcare access may be influenced by social supports, in turn impacting one’s ability to seek and access medical services. The total scale indicates good internal reliability (α = 0.89) and short-form versions have shown correlation with the long-form SSQ (Monteiro, 2011). Satisfaction with social support is scored on a six-point Likert-type scale ranging from ‘very dissatisfied’ to ‘very satisfied’. Lower score indicates less satisfaction with social support.

### Activities, participation and quality of life

#### World Health Organisation Disability Assessment Schedule (WHODAS II)

The WHODAS II (Luciano et al., 2010) questionnaire assesses overall functioning and disability and provides a global disability score. We used the 12-item version in this study. The measurement of disability is important for identifying and addressing functioning needs across different populations, including autistic adults (Park et al., 2019). Survey data indicate high internal consistency with α ranging from 0.90 to 0.97, moderate-to-good test–retest reliability and good concurrent validity in a variety of contexts (Sousa et al., 2010). Items are scored by selecting ‘none’, ‘mild’, ‘moderate’, ‘severe’ or ‘extreme’. Higher scores indicate a greater level of disability.

#### Data analysis

We conducted a cross-sectional analysis which allowed us to answer our research questions, though limited the interpretation of factors to associations rather than causes. We approached the data from a subtle realism perspective, in that self-reported perceived barriers will still prevent people from accessing services, even if that barrier objectively does not exist. Descriptive statistics were used to describe the sample and barriers to healthcare. The mean number of barriers to healthcare reported by autistic adults was compared to the mean number reported by non-autistic adults using independent samples *t*-tests. Possible predictors of barriers to healthcare from our data were identified through literature, logic or clinical expertise a priori.

A correlation matrix was used to examine associations between continuous variables to assess possible collinearity prior to regression modelling. Linear regression controlling for significant variables determined the final model of factors influencing barriers to healthcare in Australian autistic adults, with significance set at 05. All data were analysed using statistical analysis software STATA version 15. We did not adjust for multiple comparisons in order to reduce potential type 2 errors, all statistical comparisons that were conducted were planned and we avoided conducting excess comparisons beyond the research questions (Rothman, 1990).

#### Community involvement

An inclusive approach to research was adopted for this project. The non-autistic researchers consulted via email with the ALSAA RAN. The ALSAA RAN comprised of nine members, eight autistic adults and one carer at the time of consultation. The ALSAA RAN provided feedback from an autistic perspective on the research question, aims and interpretation of data, prior to commencement, once data were released and before manuscript writing. Four written responses from RAN members were received for the first review request, pertaining to research questions and aims. A further four responses were received for the second request regarding interpretation of findings.

## Results

Of 519 total self-report responses, 74 participants did not have complete responses to the BHC, with another 106 missing data on other variables, and were excluded from further analyses. Five additional autistic adults (one self-identifying, four formally diagnosed) were removed who scored <65 of the AQ-Short (Bal & Taylor, 2019), leaving *n* = 263 autistic and *n* = 70 non-autistic adults (*N* = 333). Of the autistic adults, *n* = 38 (14%) were self-identifying, with all others reporting a formal diagnosis. Regarding missing data, for autistic and non-autistic ALSAA participants, there were no differences in age, gender, AQ-Short score or geographical remoteness between those who did or did not complete the BHC or all variables in this study. Although not specifically a matched sample, mean age between autistic (43.38 years) and non-autistic (43.11 years) participants was similar (see Table 1). The majority of total participants identified as female gender (*n* = 205, 61.56%). Males represented 32.13% of the total sample (*n* = 107) and gender-diverse (non-binary, transgender, intersex) accounted for 6.31% (*n* = 21). Close to three-quarters of the sample (73.27%) resided in major cities of Australia.

**Table 1.** Participant demographics (*N* = 333).

Variable	Autistic (*n* = 263)		Non-autistic (*n* = 70)	
Age (*SD*)	43.38 (11.80)		43.11 (13.32)	
Gender (%)				
Male	91 (34.60)		16 (22.86)	
Female	151 (57.41)		54 (77.14)	
Gender-diverse^a^	21 (7.98)		0 (0)	
Ethnicity (%)				
Caucasian	184 (69.96)		39 (55.71)	
Asian	4 (1.52)		5 (7.13)	
Other	7 (2.65)		1 (1.43)	
Missing	58 (22.05)		25 (35.71)	
Remoteness (%)				
Major cities	185 (70.34)		59 (84.29)	
Inner regional	57 (21.67)		10 (14.29)	
Outer regional	16 (6.08)		1 (1.43)	
Remote	3 (1.14)		0 (0)	
Very remote	2 (0.76)		0 (0)	
Marital status (%)				
Single	78 (29.66)		13 (18.57)	
Married	82 (31.18)		39 (55.71)	
De facto	31 (11.79)		8 (11.43)	
Never married	10 (3.80)		1 (1.43)	
Widowed	1 (0.38)		2 (2.86)	
Divorced now single	28 (10.65)		1 (1.43)	
Separated	13 (4.94)		3 (4.29)	
Divorced now remarried/de facto	7 (2.66)		0 (0)	
Other	13 (4.94)		3 (4.29)	
Living status (%)				
Living alone	66 (25.10)		9 (12.86)	
Living as couple	126 (47.91)		49 (70.00)	
Living with parents	28 (10.65)		3 (4.29)	
Living with other relatives	11 (4.18)		1 (1.43)	
Living with others	25 (9.51)		6 (8.57)	
Other	7 (2.66)		2 (2.86)	
Mental health (%)	Current	Anytime	Current	Anytime
Depression	126 (47.91)	212 (80.61)	15 (21.43)	31 (44.29)
Anxiety	152 (57.79)	208 (79.08)	15 (21.43)	30 (42.86)
Social anxiety	82 (31.18)	107 (40.69)	4 (5.71)	9 (12.85)
Agoraphobia	14 (5.32)	26 (9.88)	4 (5.71)	6 (7.14)
Posttraumatic stress disorder	53 (20.15)	76 (30.04)	3 (4.29)	10 (14.29)
Obsessive compulsive disorder	23 (8.75)	34 (12.93)	0 (0)	0 (0)
Eating disorder	10 (3.80)	30 (11.40)	0 (0)	3 (4.29)
Bipolar disorder	8 (3.04)	26 (9.88)	3 (4.29)	3 (4.29)
Schizophrenia	4 (1.52)	8 (3.04)	0 (0)	0 (0)
Substance abuse or dependence	8 (3.04)	25 (9.50)	0 (0)	2 (2.86)
Attention deficit/hyperactivity disorder (ADHD)	45 (17.11)	59 (22.33)	2 (2.86)	3 (4.92)
Personality disorder	3 (1.13)	27 (10.27)	1 (1.43)	1 (1.43)
Tic disorders (e.g. Tourette’s syndrome)	2 (0.76)	4 (1.52)	0 (0)	0 (0)
Any other mental health disorder	17 (6.44)	22 (8.33)	0 (0)	0 (0)

SD: standard deviation.

a Gender-diverse = non-binary, transgender, intersex.

All participants in the study are represented by a total score between 0 and 17 on the BHC. Table 2 compares scores for each item of the BHC between autistic and non-autistic adults. Over three-quarters of autistic participants (*n* = 210, 79%) reported at least one or more barriers, with almost one in five (*n* = 70, 19%) reporting eight or more barriers within the BHC. Over three-quarters of non-autistic participants reported zero barriers (*n* = 73, 6.04%). The mean score of barriers to healthcare experienced by the autistic adults was 4.58 and significantly higher than the 0.76 mean barriers experienced by non-autistic adults (*M* difference: 3.83, *t*_347_ = 8.92, *p* ⩽ 0.001, 95% CI = 2.98–4.67). Some of the main barriers which were heavily endorsed by autistic people and rarely endorsed by non-autistic people include handling the waiting room, following-up on care and identifying or reporting pain and physical symptoms to healthcare providers.

**Table 2.** Barriers to Healthcare Checklist Short-Form item-level responses between autistic and non-autistic participants, number of people who answered yes (*N* = 333).

Item	Autistic (*n* = 263)	Non-autistic (*n* = 70)
	*n* (*%*)	*n* (*%*)
Fear, anxiety, embarrassment or frustration keeps me from getting primary car	103 (39.16)	10 (14.29)
I have trouble following up on care	104 (39.54)	4 (5.71)
I have difficulty understanding how to translate medical information into concrete steps that I can take to improve my health	50 (19.02)	0 (0)
I don’t understand the healthcare system	44 (16.73)	0 (0)
It is too difficult to make appointments	76 (28.90)	9 (12.86)
I have problems filling out paperwork	36 (13.69)	0(0)
My behaviours are misinterpreted by my provider or staff	79 (30.04)	1 (1.43)
My providers or the staff do not take my communications seriously	72 (27.38)	2 (2.86)
I cannot find a healthcare provider who will accommodate my needs	65 (24.71)	4 (5.71)
My providers or the staff do not include me in discussions about my health	24 (9.13)	0 (0)
Communication with my healthcare provider or the staff is too difficult	66 (25.10)	3 (4.29)
When I experience pain and/or other physical symptoms, I have difficulties identifying them and reporting them to my healthcare provider	107 (40.68)	4 (5.71)
Sensory discomforts get in the way of my healthcare	86 (32.70)	1 (1.43)
Concerns about cost or insurance coverage keep me from getting primary care	118 (44.87)	10 (14.29)
I do not have a way to get to my doctor’s office	8 (3.04)	1 (1.43)
I have inadequate social, family or caregiver support	57 (21.67)	4 (5.71)
I find it hard to handle the waiting room	111 (42.21)	1 (1.43)

Scores for the BHC and each of the predictor variables for autistic and non-autistic participants (see Table 3) highlight key differences in all categories, with autistic participants scoring higher on average in each measure.

**Table 3.** Characteristics of possible predictor measures for autistic and non-autistic participants (*N* = 333).

Item	Autistic (*n* = 263)	Non-autistic (*n* = 70)
	*M* (*SD*)	*M* (*SD*)
AQ-Short (Autism Severity)	88.95 (9.84)	56.04 (12.11)
PHQ-15 (Somatic Symptom Severity)	10.12 (5.11)	5.31 (3.05)
PHQ-9 (Depression)	11.38 (7.2)	5.13 (5.46)
SF-12 PCS (Physical Health)	44.85 (12.37)	50.24 (8.09)
GAD-A (Anxiety)	13.81 (8.02)	5.5 (5.52)
ULS-8 (Loneliness)	22.9 (5.16)	16.41 (5.77)
WHODAS-II (Disability Score)	26.19 (8.74)	15.44 (4.7)
SSQ (Social Support Satisfaction)	16.95 (9.12)	11.36 (6.89)

SD: standard deviation; PHQ: Patient Health Questionnaire; PCS: Physical Component Scale; GAD-A: Generalised Anxiety Disorder–Adult; ULS: UCLA Loneliness Scale; WHODAS-II: World Health Organisation Disability Assessment Schedule II; SSQ: Social Support Questionnaire.

Table 4 shows pairwise correlations for predictor variables included in the final analysis. The majority of variables showed moderate-to-strong correlations. These findings suggest that increased autism symptoms, increased somatic symptoms, increased depression, poorer physical health, increased anxiety, increased loneliness, greater disability and decreased satisfaction with social support contribute to more barriers to healthcare access.

**Table 4.** Pairwise correlations autistic sample (*n* = 263).

Variables	(1)	(2)	(3)	(4)	(5)	(6)	(7)	(8)	(9)
(1) BHC	1.00								
(2) AQ-Short (Autism Symptoms)	.27***	1.00							
(3) PHQ-15 (Somatic Symptom Severity)	.44***	.14**	1.00						
(4) PHQ-9 (Depression)	.46***	.27***	.44***	1.00					
(5) SF-12 PCS (Physical Health)	−.27***	−.02	−.45***	−.13**	1.00				
(6) GAD-A (Anxiety)	.48***	.31***	.47***	.74***	−.10	1.00			
(7) ULS-8 (Loneliness)	.36***	.29***	.22***	.45***	−.07	.35***	1.00		
(8) WHODAS II (Disability Score)	.59***	.30***	.55***	.59***	−.51***	.59***	.35***	1.00	
(9) SSQ (Satisfaction with Social Support)	.32***	.13**	.20***	.39***	−.15**	.26***	.58***	.24***	1.00

BHC: Barriers to Healthcare Checklist; PHQ: Patient Health Questionnaire; PCS: Physical Component Scale; GAD-A: Generalised Anxiety Disorder–Adult; ULS: UCLA Loneliness Scale; WHODAS-II: World Health Organisation Disability Assessment Schedule II; SSQ: Social Support Questionnaire;

*** *p* < 0.01; **p < 0.05.

Entering all predictors into a regression model (see Table 5) found autistic adults who identified as gender-diverse, reported higher levels of anxiety (GAD-A), greater levels of disability (WHODAS II) or less satisfaction with social support (SSQ satisfaction score) were likely to experience more barriers to accessing healthcare as reported by the BHC. The final model had a high amount of variance explained (adj. *R*^2^ = 0.46).

**Table 5.** Regression model predicting barriers to healthcare total score (*n* = 263).

BHC	Coef.	*SE*	*t*-value	[95% CI]		*p*
Age	−0.02	0.02	−1.42	−0.06	0.01	0.158
Gender						
Male		–	–	–	–	–
Female	0.17	0.43	0.40	−0.68	1.02	0.691
Gender-diverse^a^	2.2	0.81	2.72	0.60	3.80	0.007**
Remoteness						
Major cities		–	–	–	–	–
Inner regional	−0.58	0.49	−1.19	−1.55	0.38	0.236
Other	0.27	0.75	0.36	−1.21	1.75	0.721
AQ (Autism Severity)	0.03	0.02	1.19	−0.02	0.07	0.236
PHQ-15 (Somatic Symptom Severity)	0.06	0.05	1.27	−0.03	0.16	0.204
PHQ-9 (Depression)	−0.00	0.04	−0.10	−0.09	0.08	0.92
SF-12 PCS (Physical Health)	0.02	0.02	0.78	−0.03	0.06	0.43
GAD (Anxiety)	0.09	0.04	2.12	0.01	0.17	0.03**
ULS-8 (Loneliness)	0.03	0.05	0.54	−0.07	0.13	0.59
WHODAS-II (Disability Score)	0.18	0.04	4.91	0.11	0.25	<0.001***
SSQ (Satisfaction with Social Support)	0.07	0.03	2.60	0.02	0.12	0.01**
Constant	−5.95	2.47	−2.41	−10.81	−1.08	0.02**
*R* ^2^		.46				
*F*-test		16.12	Prob > *F*	<.001***		

BHC: Barriers to Healthcare Checklist; AQ: Autism Quotient; PHQ: Patient Health Questionnaire; PCS: Physical Component Scale; GAD: Generalised Anxiety Disorder; ULS: UCLA Loneliness Scale; WHODAS-II: World Health Organisation Disability Assessment Schedule II; SSQ: Social Support Questionnaire;

a Gender-diverse = non-binary, transgender, intersex

** *p* < 0.05; ***p < 0.001.

## Discussion

This study adds substantially to the body of knowledge about barriers to healthcare using the BHC. Key findings suggest Australian autistic adults experience more barriers to healthcare than their non-autistic counterparts, and although we did not investigate underlying mechanisms, gender diversity, anxiety, social support and level of disability were associated with increased perceived barriers for autistic adults. Our findings demonstrate a positive association between higher levels of disability in autistic adults as scored on WHODAS-II (Sousa et al., 2010) and the numbers of barriers reported on the BHC. Further positive associations were found between greater severity of anxiety as measured by the *DSM*-5 GAD-A (Beesdo-Baum, Klotsche et al., 2012), less satisfaction with social support as measured by the SSQ and the number of barriers reported on the BHC by autistic adults. Our findings suggest autistic adults who identify as gender-diverse experience greater barriers to healthcare. To our knowledge, this is the first study to identify gender diversity associated with barriers to accessing healthcare in an autistic cohort. These findings align with previous research, conducted in other parts of the world, highlighting barriers to healthcare for autistic adults such as ineffective communication and high rates of co-occurring health conditions (Hirvikoski et al., 2016; Matson, 2016; Nicolaidis et al., 2013, 2014, 2015; Raymaker et al., 2017). These barriers existing in the modern Australian health system have been identified in international samples and are likely exacerbated in some countries.

The significant relationship identified between anxiety and healthcare barriers in our autistic adult sample has also been a focus of research efforts in recent history in non-autistic populations. Generalised anxiety disorder (GAD) has been associated with reassurance-seeking health behaviours or avoidance of care (Beesdo-Baum, Jenjahn et al., 2012). Research into this relationship by Horenstein and Heimberg (2020) suggests people with GAD predominantly demonstrate reassurance-seeking behaviours; however, less research has been conducted on avoidance behaviours. Although individuals with GAD typically require a higher level of care from primary healthcare institutions, they experience greater barriers when accessing these services and report significant challenges in patient–physician-related factors and societal barriers such as stigma (Davidson et al., 2010). The challenges associated with accessing healthcare are further complicated when considering autistic adults. Feedback from the ALSAA RAN suggested autistic adults with co-occurring GAD may demonstrate behavioural avoidance of anxiety inducing stimuli, which may be influenced by past negative experiences when accessing healthcare. For example, experiences where autistic adults with GAD have not been believed or have had their concerns dismissed by health professionals. Autistic adults experience high rates of co-occurring anxiety, which is associated with excessive worrying over health outcomes (Brondino et al., 2019). Evidence suggests that worry is associated with diminished emotional responses and therefore may be a source of avoidance in individuals with GAD (Behar et al., 2009). For autistic adults, anxiety and worry may be further exacerbated by the complexities of managing multiple co-occurring conditions and the differing health services related to each of them. This avoidance type behaviour may cause barriers to both seeking and accessing care which can impact negatively on health outcomes.

Our study identified a positive association between greater disability severity as measured by the WHODAS-II and number of identified barriers to healthcare on the BHC. These findings may suggest that healthcare facilities are ill equipped to meet the demands of individuals with significant disability, with the Australian Disability Royal Commission reporting systematic neglect of people with cognitive disability in the Australian health system (Royal Commission into Violence, Abuse, Neglect and Exploitation of People with Disability, 2020). Previous research investigating the relationship between adults with disability accessing healthcare supports this assertion, for example, accounts of autistic adults and people with disability being apprehensive to seek healthcare due to their health needs or disability being misunderstood (e.g. Bradshaw et al., 2019; Drainoni et al., 2006; Gerber et al., 2017; Iacono et al., 2014; Kang et al., 2022). Our findings add to the weight of evidence highlighting the need for reform of health systems to be accessible to autistic adults and other people with disability with higher levels of support needs.

The relationship we found between anxiety, level of disability (as measured by the WHODAS-II) and healthcare barriers, may stem from autism often being a hidden disability, not always immediately apparent to others. When a disability is not clearly visible assumptions may be made regarding the functional ability of an individual. This can lead to poor patient–provider communication, an integral element of successful healthcare experiences (David & Gerhard, 2017; Patak et al., 2009). Nicolaidis et al. (2015) found many autistic participants attributed negative experiences with healthcare access to incorrect assumptions about individuals’ skills or needs. Such assumptions can be either an over- or underassumption of an individual’s functional capabilities. A lack of physician knowledge and training on treating autistic patients exacerbates this issue and is reported repeatedly by autistic people (Bruder et al., 2012; Mason et al., 2019). Providers report difficulty with establishing rapport and implementing appropriate communication strategies with their autistic patients (Zerbo et al., 2015). If healthcare providers are unaware of what it means to be autistic, they may be unaware of how to adapt treatment strategies for autistic people. As suggested by participants from the Nicolaidis et al. (2015) study, providers may attribute behaviours to autistic symptoms rather than the presence of an illness in a process known as diagnostic overshadowing.

Analogous to our findings, social support is intrinsically linked to well-being and health in the general population (Mazurek, 2014). Although there is limited research available on loneliness in autistic adults, studies on autistic children have reported greater loneliness than typically developing children (Bauminger et al., 2003; Locke et al., 2010). Ee et al. (2019) reported autistic adults scored significantly higher on the ULS-8 loneliness measurement than non-autistic participants, with the presence of autism contributing to the greatest variance in loneliness score. The presence of loneliness indicates lesser available social support, which may have negative implications on healthcare access. As suggested by the ALSAA RAN, autistic adults may rely on social supports to identify experiences of poor health and prompt them to seek healthcare. Similarly, Nicolaidis et al. (2014) note cognitive differences in autistic adults can impact their ability to identify and manage illnesses. For autistic adults experiencing co-occurring conditions or a greater level of disability, they may also require a support person to assist them in appointment management and transport to healthcare facilities. Tobin et al. (2014) found autistic adults often do not have highly populous social networks, but the perceived ability of this social network to provide support is more important than the number of people within the network. Within our study autistic adults who reported dissatisfaction with available social supports, also perceived greater barriers to healthcare. Similarly, Havercamp et al. (2004) investigated health disparities experienced by adults with developmental disabilities and reported a correlation between lack of social support with poorer overall quality of life and mental health outcomes.

In this study, a positive correlation was identified between barriers reported on the BHC and participants who reported their gender as gender-diverse. The ‘gender-diverse’ category within this study included those who did not identify with the gender they were assigned at birth, intersex, non-binary and transgender participants. In Western countries including Australia, gender identity is presumed to align with assigned sex at birth (Cislaghi & Heise, 2020). One study by Warrier et al. (2020) found within their sample that transgender and gender-diverse people were 3.03 to 6.36 times as likely to be autistic than cisgender individuals (identifying with the sex assigned at birth); however, this increased rate of gender diversity is not specific only to autism. In recent history, there has been increasing focus on the role of medical practitioners within Australia providing health services to gender-diverse people (Riggs et al., 2014). As Riggs et al. explain, positive experiences with accessing healthcare of gender-diverse people are rare. Experiences of gender-diverse people being denied services, encountering practitioners with little to no knowledge on treating gender-diverse people, experiencing increased waiting times, being subjected to the use of inappropriate terminology and having their own knowledge discounted, are more common. These findings suggest that gender-diverse people experience ongoing barriers and disparities with accessing healthcare, which align with our findings of autistic gender-diverse people. Systemic barriers including transphobia perpetuate these barriers and may deter gender-diverse people from accessing healthcare (Goldstein et al., 2017). Autistic adults who are gender-diverse experience a double disadvantage when accessing healthcare, as they not only experience barriers related to autism, but also related to contradicting the norms of a heteronormative society (Bosse, 2019).

### Implications for practice

Autistic adults currently experience barriers to accessing healthcare in Australia, and internationally. To mitigate barriers faced by autistic adults, new policies and practices, co-designed and co-developed with autistic people and other stakeholders, are needed. By co-design we refer to processes that engage end-users in a collaborative partnership with practitioners or researchers (Taylor et al., 2022). Involving autistic people in the development of policies and practices will allow targeted strategies to support healthcare delivery, reduce barriers to healthcare and ensure healthcare needs are met in a timely, respectful and person-centred way. Particular actions may include post-diagnosis services, supports initiatives for autistic adults, actions to reduce cost of diagnosis, community acceptance initiatives, training for clinicians on neurodiversity and heterogenic presentations or autism (Huang et al., 2022), clinical environment accommodations, targeted funding (den Houting & Pellicano, 2019) and research particularly on the high rates of co-occurring health and mental health conditions and the development of clinical care pathways. Recently, a National Roadmap for Improving the Health of People with Intellectual Disability (Department of Health, 2021) was released in Australia. A similar national roadmap is needed to address the current unmet health needs of autistic adults. Roadmaps such as these identify and implement specific models of care to support healthcare practitioners to deliver quality care, and ensure that the health needs of the target population are met. We are hopeful that recent moves towards an Australian national autism strategy (Autism CRC, 2022) includes mandates to improve service delivery, as does the United Kingdom Autism Act (All Party Parliamentary Group on Autism, 2019).

In the delivery of healthcare to autistic people, environmental adaptations could be implemented to reduce barriers to healthcare. Example interventions include allowing extra time for an appointment, reducing sensory stimuli and delivering health-related information in an accessible way for autistic people (Nicolaidis et al., 2014). Strategies such as allowing additional time, providing detailed explanations of what will occur during a healthcare visit and warning the patient before physically examining them are included recommendations in the Academic Autism Spectrum Partnership in Research and Education healthcare toolkit (Nicolaidis et al., 2016). This toolkit has demonstrated benefits for autistic adults in healthcare settings, and such strategies would be appropriate to adapt to Australian context (Kang et al., 2022). Using alternative communication such as visual aids and assistive devices to improve patient–provider communication may reduce barriers experienced by autistic adults when accessing healthcare (Collier et al., 2010; Drainoni et al., 2006).

Practitioner training is another key component to accessible healthcare for autistic adults and should occur prior to entry into the workforce. Practitioner education needs to address knowledge gaps in health professionals on autism, disability and how to relate to autistic people in healthcare contexts and inclusive curriculum development and educational approaches are key. Training to improve healthcare for gender-diverse people is also needed. The ALSAA RAN suggested taking a pro-active approach to training health professional training will limit the passive influence of ignorance on these matters. Such training will play a role in tackling the broader systemic barriers facing gender-diverse people (Rider et al., 2019). Improving healthcare access is also contingent on improving social supports available for autistic adults. Connecting autistic adults to appropriate social support may improve quality of life and reduce some of the associated barriers with accessing healthcare (Tobin et al., 2014).

### Strengths and limitations

A key strength of the study was collection of data from a large nationwide sample; however, some limitations must be noted. Although the survey could be completed self-paced across multiple sessions, the length of the ALSAA survey likely was a barrier to some potential participants and likely also affected the amount of missing data. Despite nationwide sampling, autistic adults with intellectual disability were underrepresented, and as reported elsewhere (Arnold, Foley et al., 2019; Harvery et al., 2021), our sample had slightly above average education and socioeconomic status. However, this highlights that autistic adults even in less-disadvantaged circumstances experience significant barriers to access healthcare. Although autistic adults in our sample reported very high rates of mental illness as is noted in the literature, non-autistic participants also reported somewhat elevated rates in comparison to Australia population figures, which may suggest self-selection bias for the non-autistic sample, though again highlights the great increase in barriers experienced by our autistic compared to non-autistic participants. The survey requiring sufficient English language skills also affected sampling, with ethnicity appearing slightly less diverse than the Australian population. It is likely that people without English language skills would experience compounding barriers in the Australia healthcare system, and these Australian autistic adults have not been represented in our study.

This study included both participants who have been formally diagnosed with autism, and those who have not been diagnosed, however identify as being autistic. Prior to improvements in diagnostic tools and greater autism knowledge, many autistic adults went undiagnosed as children and remain undiagnosed in adulthood (Evans, 2013; Lai & Baron-Cohen, 2015). For this reason, those who have not been formally diagnosed were invited to participate in the study, though only constituted a small proportion of the sample. Future studies should consider that self-identifying autistic adults may not have the same legal basis to reasonable adjustments in accessing healthcare, which brings into consideration universal design versus specific accommodations. While quantitative data allowed researchers to determine associations between predictors and healthcare access barriers in a large sample of autistic adults, qualitative data may provide rich information and insight into the lived experience of autistic adults accessing healthcare. Qualitative studies are important to further this research and our understanding of the difficulties faced by autistic adults. Overall, our findings could provide helpful insights for conducting a thorough realist evaluation into barriers to healthcare for autistic adults.

The majority of participants within this study identified as female. Frequently autism survey research has demonstrated a ‘reversed sex-ratio’ where female participants are overrepresented (Rødgaard et al., 2022). It has been suggested that autistic females experience autistic symptoms which are not represented in the current diagnostic criteria and therefore there may be more autistic females than previously realised. Another possible reason for the discrepancy between genders is that females are more likely to respond to surveys (Arnold, Foley et al., 2019). Overall, results must be interpreted with consideration of the sample gathered, and particularly may not be easily generalised to autistic males. Future research particularly should attempt to sample autistic adults with intellectual disability and those less likely to engage in primarily online survey research. Finally, although an inclusive research approach including consultation with autistic adults was employed, ideally future research will utilise autistic researchers and peer researchers, as co-production may further improve the depth, understanding and nuance of interpretations (den Houting et al., 2022).

## Conclusion

This study found Australian autistic adults experience greater barriers to healthcare than Australian non-autistic adults. Greater disability, higher levels of anxiety, less satisfaction with social supports and gender diversity were associated with greater barriers to accessing healthcare in our sample of Australian autistic adults. Our findings highlight the need for interventions to assist healthcare facilities in meeting the needs of autistic individuals. Such interventions may include the introduction of a roadmap for improving healthcare for autistic adults, as well as new policies to advocate for the rights of people with a disability. The findings of this study also demonstrate a need for further research into the experiences of autistic adults with healthcare, in a field which is dominated by paediatric research efforts. Further research in an Australian context is also required, with most knowledge to date generated in healthcare systems in other jurisdictions, which have important differences to the Australian environment.

## References

[bibr1-13623613231168444] All Party Parliamentary Group on Autism. (2019). The autism act. 10 years on: A report from the all party parliamentary group on autism on understanding, services and support for autistic people and their families in England. https://www.autism.org.uk/what-we-do/campaign/not-enough/about-the-autism-act

[bibr2-13623613231168444] Arnold S. R. C. Foley K.-R. Hwang Y. I. Richdale A. L. Uljarevic M. Lawson L. P. Cai R. Y. Falkmer T. Falkmer M. Lennox N. G. Urbanowicz A. Trollor J. (2019). Cohort profile: The Australian Longitudinal Study of Adults with Autism (ALSAA). British Medical Journal Open, 9(12), Article e030798. 10.1136/bmjopen-2019-030798 PMC6924702 31806608

[bibr3-13623613231168444] Arnold S. R. C. Higgins J. M. Trollor J. N. (2020). Health services for Australian autistic adults: Commentary on ‘The experiences, views, and needs of health professionals who provide care to adults on the autism spectrum’ (Urbanowicz, Parkin, van Dooren, Girdler, Ciccarelli, & Lennox, 2020). Research and Practice in Intellectual and Developmental Disabilities, 7(2), 193–198. 10.1080/23297018.2020.1753566

[bibr4-13623613231168444] Arnold S. R. C. Uljarević M. Hwang Y. I. Richdale A. L. Trollor J. N. Lawson L. P. (2019). Brief report: Psychometric properties of the Patient Health Questionaire-9 (PHQ-9) in autistic adults. Journal of Autism and Developmental Disorders, 50(6), 2217–2225. 10.1007/s10803-019-03947-9 30847710

[bibr5-13623613231168444] Australian Bureau of Statistics. (2020). Australian Statistical Geography Standards (ASGS) edition 3. https://www.abs.gov.au/statistics/standards/australian-statistical-geography-standard-asgs-edition-3/jul2021-jun2026

[bibr6-13623613231168444] Autism Cooperative Research Centre. (2022). Funding to support national autism strategy development. https://www.autismcrc.com.au/news/latest-news/funding-support-national-autism-strategy-development

[bibr7-13623613231168444] Bal V. H. Taylor J. L. (2019). Advancing understanding of adults: The role of diagnostic confirmation and sample description. Autism, 23(4), 807–810. 10.1177/1362361319847547 31044599

[bibr8-13623613231168444] Baron-Cohen S. Wheelwright S. Skinner R. Martin J. Clubley E. (2001). The Autism-Spectrum Quotient (AQ): Evidence from Asperger syndrome/high-functioning autism, males and females, scientists and mathematicians. Journal of Autism and Developmental Disorders, 31(1), 5–17. 11439754 10.1023/a:1005653411471

[bibr9-13623613231168444] Bauminger N. Shulman C. Agam G. (2003). Peer interaction and loneliness in high-functioning children with autism. Journal of Autism and Developmental Disorders, 33(5), 489–507. 10.1023/A:1025827427901 14594329

[bibr10-13623613231168444] Beesdo-Baum K. Jenjahn E. Höfler M. Lueken U. Becker E. S. Hoyer J. (2012). Avoidance, safety behavior, and reassurance seeking in generalized anxiety disorder. Depression and Anxiety, 29(11), 948–957. 10.1002/da.21955 22581482

[bibr11-13623613231168444] Beesdo-Baum K. Klotsche J. Knappe S. Craske M. G. LeBeau R. T. Hoyer J. Strobel A. Pieper L. Wittchen H.-U. (2012). Psychometric properties of the dimensional anxiety scales for DSM-V in an unselected sample of German treatment seeking patients. Depression and Anxiety, 29(12), 1014–1024. 10.1002/da.21994 22933460

[bibr12-13623613231168444] Behar E. DiMarco I. D. Hekler E. B. Mohlman J. Staples A. M. (2009). Current theoretical models of generalized anxiety disorder (GAD): Conceptual review and treatment implications. Journal of Anxiety Disorders, 23(8), 1011–1023. 10.1016/j.janxdis.2009.07.006 19700258

[bibr13-13623613231168444] Bergman M. A. Schene A. H. Vissers C. Th. W. M. Vrijsen J. N. Kan C. C. van Oostrom I. (2020). Systematic review of cognitive biases in autism spectrum disorders: A neuropsychological framework towards an understanding of the high prevalence of co-occurring depression. Research in Autism Spectrum Disorders, 69(8), 101455. 10.1016/j.rasd.2019.101455

[bibr14-13623613231168444] Bosse J. D. (2019). Sexual and gender identity development in young adults and implications for healthcare. Current Sexual Health Reports, 11(4), 274–286. 10.1007/s11930-019-00215-w

[bibr15-13623613231168444] Bradshaw P. Pellicano E. van Driel M. Urbanowicz A. (2019). How can we support the healthcare needs of autistic adults without intellectual disability? Current Developmental Disorders Reports, 6(2), 45–56. 10.1007/s40474-019-00159-9

[bibr16-13623613231168444] Brondino N. Fusar-Poli L. Miceli E. Di Stefano M. Damiani S. Rocchetti M. Politi P. (2019). Prevalence of medical comorbidities in adults with autism spectrum disorder. Journal of General Internal Medicine, 34(10), 1992–1994. 10.1007/s11606-019-05071-x 31144278 PMC6816725

[bibr17-13623613231168444] Bruder M. B. Kerins G. Mazzarella C. Sims J. Stein N. (2012). Brief report: The medical care of adults with autism spectrum disorders: Identifying the needs. Journal of Autism and Developmental Disorders, 42(11), 2498–2504. 10.1007/s10803-012-1496-x 22427260

[bibr18-13623613231168444] Callander E. J. Fox H. Lindsay D. (2019). Out-of-pocket healthcare expenditure in Australia: Trends, inequalities and the impact on household living standards in a high-income country with a universal health care system. Health Economics Review, 9(1), 1–8. 10.1186/s13561-019-0227-9 30859357 PMC6734455

[bibr19-13623613231168444] Calleja S. Islam F. Kingsley J. McDonald R. (2019). The disparities of healthcare access for adults with autism spectrum disorder. Medicine, 98(7), Article e14480. 10.1097/md.0000000000014480 PMC6408059 30762771

[bibr20-13623613231168444] Cameron I. M. Crawford J. R. Lawton K. Reid I. C. (2008). Psychometric comparison of PHQ-9 and HADS for measuring depression severity in primary care. British Journal of General Practice, 58(546), 32–36. 10.3399/bjgp08X263794 PMC2148236 18186994

[bibr21-13623613231168444] Cislaghi B. Heise L. (2020). Gender norms and social norms: Differences, similarities and why they matter in prevention science. Sociology of Health & Illness, 42(2), 407–422. 10.1111/1467-9566.13008 31833073 PMC7028109

[bibr22-13623613231168444] Collier B. McGhie-Richmond D. Self H. (2010). Exploring communication assistants as an option for increasing communication access to communities for people who use augmentative communication. Augmentative and Alternative Communication, 26(1), 48–59. 10.3109/07434610903561498 20196704

[bibr23-13623613231168444] Croen L. A. Zerbo O. Qian Y. Massolo M. L. Rich S. Sidney S. Kripke C. (2015). The health status of adults on the autism spectrum. Autism, 19(7), 814–823. 10.1177/1362361315577517 25911091

[bibr24-13623613231168444] David R. Gerhard S. (2017). The influence of doctor-patient communication on health outcomes: A systematic review. Zeitschrift für Psychosomatische Medizin und Psychotherapie, 63(2), 131–150. 10.13109/zptm.2017.63.2.131 28585507

[bibr25-13623613231168444] Davidson J. R. T. Feltner D. E. Dugar A. (2010). Management of generalized anxiety disorder in primary care: Identifying the challenges and unmet needs. Primary Care Companion to the Journal of Clinical Psychiatry, 12(2). Article 772. 10.4088/PCC.09r00772blu PMC2911006 20694114

[bibr26-13623613231168444] den Houting J. Higgins J. Isaacs K. Mahony J. Pellicano E . (2022). From ivory tower to inclusion: Stakeholders’ experiences of community engagement in Australian autism research. Frontiers in Psychology, 13, Article 876990. 10.3389/fpsyg.2022.876990 PMC9454607 36092113

[bibr27-13623613231168444] den Houting J. Pellicano E . (2019). A portfolio analysis of autism research funding in Australia, 2008–2017. Journal of Autism and Developmental Disorders, 49(11), 4400–4408. 10.1007/s10803-019-04155-1 31375971

[bibr28-13623613231168444] Department of Health. (2021). National roadmap for improving the health of people with intellectual disability. https://www.health.gov.au/resources/publications/national-roadmap-for-improving-the-health-of-people-with-intellectual-disability

[bibr29-13623613231168444] Dixit S. K. Sambasivan M. (2018). A review of the Australian healthcare system: A policy perspective. SAGE Open Medicine, 6, Article 11876921. 10.1177/2050312118769211 PMC5900819 29686869

[bibr30-13623613231168444] Drainoni M.-L. Lee-Hood E. Tobias C. Bachman S. S. Andrew J. Maisels L. (2006). Cross-disability experiences of barriers to health-care access: Consumer perspectives. Journal of Disability Policy Studies, 17(2), 101–115. 10.1177/10442073060170020101

[bibr31-13623613231168444] Ee D. Hwang Y. I. Reppermund S. Srasuebkul P. Troller J. N. Foley K.-R. Arnold S. R. C. (2019). Loneliness in adults on the autism spectrum. Autism in Adulthood, 1(3), 182–193. 10.1089/aut.2018.0038 36601420 PMC8992830

[bibr32-13623613231168444] Evans B. (2013). How autism became autism: The radical transformation of a central concept of child development in Britain. History of the Human Sciences, 26(3), 3–31. 10.1177/0952695113484320 24014081 PMC3757918

[bibr33-13623613231168444] Frieling M. A. Davis W. R. Chiang G. (2013). The SF-36v2 and SF-12v2 health surveys in New Zealand: Norms, scoring coefficients and cross-country comparisons. Australian and New Zealand Journal of Public Health, 37(1), 24–31. 10.1111/1753-6405.12006 23379802

[bibr34-13623613231168444] Gerber A. H. McCormick C. E. B. Levine T. P. Morrow E. M. Anders T. F. Sheinkopf S. J. (2017). Brief report: Factors influencing healthcare satisfaction in adults with autism spectrum disorder. Journal of Autism and Developmental Disorders, 47(6), 1896–1903. 10.1007/s10803-017-3087-3 28271179

[bibr35-13623613231168444] Gerst-Emerson K. Jayawardhana J. (2015). Loneliness as a public health issue: The impact of loneliness on health care utilization among older adults. American Journal of Public Health, 105(5), 1013–1019. 10.2105/AJPH.2014.302427 25790413 PMC4386514

[bibr36-13623613231168444] Goldstein Z. Corneil T. A. Greene D. N. (2017). When gender identity doesn’t equal sex recorded at birth: The role of the laboratory in providing effective healthcare to the transgender community. Clinical Chemistry, 63(8), 1342–1352. 10.1373/clinchem.2016.258780 28679645

[bibr37-13623613231168444] Han C. Pae C.-U. Patkar A. A. Masand P. S. Woong Kim K. Joe S.-H. Jung I.-K. (2011). Psychometric properties of the Patient Health Questionnaire–15 (PHQ–15) for measuring the somatic symptoms of psychiatric outpatients. Psychosomatics, 50(6), 580–585. 10.1016/S0033-3182(09)70859-X 19996228

[bibr38-13623613231168444] Harvery M. Froude E. H. Foley K.-R. Trollor J. N. Arnold S. R. C. (2021). Employment profiles of autistic adults in Australia. Autism Research, 14(10), 2061–2077. 10.1002/aur.2588 34374491

[bibr39-13623613231168444] Havercamp S. M. Scandlin D. Roth M. (2004). Health disparities among adults with developmental disabilities, adults with other disabilities, and adults not reporting disability in North Carolina. Public Health Reports, 119(4), 418–426. 10.1016/j.phr.2004.05.006 15219799 PMC1497651

[bibr40-13623613231168444] Hirvikoski T. Mittendorfer-Rutz E. Boman M. Larsson H. Lichtenstein P. Bölte S. (2016). Premature mortality in autism spectrum disorder. British Journal of Psychiatry, 208(3), 232–238. 10.1192/bjp.bp.114.160192 26541693

[bibr41-13623613231168444] Hoekstra R. A. Vinkhuyzen A. A. E. Wheelwright S. Bartels M. Boomsma D. I. Baron-Cohen S. Posthuma D. van der Sluis S. (2010). The construction and validation of an abridged version of the Autism-Spectrum Quotient (AQ-Short). Journal of Autism and Developmental Disorders, 41(5), 589–596. 10.1007/s10803-010-1073-0 PMC3076581 20697795

[bibr42-13623613231168444] Horenstein A. Heimberg R. G. (2020). Anxiety disorders and healthcare utilization: A systematic review. Clinical Psychology Review, 81, Article 101894. 10.1016/j.cpr.2020.101894 32818687

[bibr43-13623613231168444] Huang Y. Arnold S. R. C. Foley K.-R. Trollor J. N. (2022). A qualitative study of adults’ and support persons’ experiences of support after autism diagnosis. Journal of Autism and Developmental Disorders. Advance online publication. 10.1007/s10803-022-05828-0 PMC9734854 36484961

[bibr44-13623613231168444] Iacono T. Bigby C. Unsworth C. Douglas J. Fitzpatrick P. (2014). A systematic review of hospital experiences of people with intellectual disability. BMC Health Services Research, 14(1), Article 505. 10.1186/s12913-014-0505-5 PMC4210514 25344333

[bibr45-13623613231168444] Kang L. R. J. Barlott T. Turpin M. Urbanowicz A. Kang L. R. J. Barlott T. Turpin M. Urbanowicz A. (2022). A trial of the AASPIRE healthcare toolkit with Australian adults on the autism spectrum. Australian Journal of Primary Health, 28(4), 350–356. 10.1071/PY21134 35550238

[bibr46-13623613231168444] Khanna R. Jariwala-Parikh K. West-Strum D. Mahabaleshwarkar R. (2014). Health-related quality of life and its determinants among adults with autism. Research in Autism Spectrum Disorders, 8(3), 157–167. 10.1016/j.rasd.2013.11.003

[bibr47-13623613231168444] Kroenke K. Spitzer R. L. Williams J. B. W. (2001). The PHQ-9: Validity of a brief depression severity measure. Journal General International Medicine, 16(9), 606–613. 10.1046/j.1525-1497.2001.016009606.x PMC1495268 11556941

[bibr48-13623613231168444] Laba T.-L. P. Usherwood T. Leeder S. Yusuf F. Gillespie J. Perkovic V. Wilson A. Jan S. Essue B. (2015). Co-payments for health care: What is their real cost? Australian Health Review, 39(1), 33–36. 10.1071/AH14087 25362348

[bibr49-13623613231168444] Lai M.-C. Baron-Cohen S. (2015). Identifying the lost generation of adults with autism spectrum conditions. The Lancet Psychiatry, 2(11), 1013–1027. 10.1016/S2215-0366(15)00277-1 26544750

[bibr50-13623613231168444] Locke J. Ishijima E. H. Kasari C. London N. (2010). Loneliness, friendship quality and the social networks of adolescents with high-functioning autism in an inclusive school setting. Journal of Research in Special Educational Needs, 10(2), 74–81. 10.1111/j.1471-3802.2010.01148.x

[bibr51-13623613231168444] Luciano J. V. Ayuso-Mateos J. L. Aguado J. Fernandez A. Serrano-Blanco A. Roca M. Haro J. M. (2010). The 12-item World Health Organization Disability Assessment Schedule II (WHO-DAS II): A nonparametric item response analysis. BMC Medical Research Methodology, 10, Article 45. https://doi.org/http://dx.doi.org/10.1186/1471-2288-10-45 10.1186/1471-2288-10-45 PMC2881065 20487526

[bibr52-13623613231168444] Malik-Soni N. Shaker A. Luck H. Mullin A. Wiley R. Lewis M. Fuentes J. Frazier T. W. (2021). Tackling healthcare access barriers for individuals with autism from diagnosis to adulthood. Pediatric Research, 91(5), 1028–1035. 10.1038/s41390-021-01465-y 33767375 PMC7993081

[bibr53-13623613231168444] Mason D. Ingham B. Urbanowicz A. Michael C. Birtles H. Woodbury-Smith M. Brown T. James I. Scarlett C. Nicolaidis C. Parr J. R. (2019). A systematic review of what barriers and facilitators prevent and enable physical healthcare services access for autistic adults. Journal of Autism and Developmental Disorders, 49(8), 3387–3400. 10.1007/s10803-019-04049-2 31124030 PMC6647496

[bibr54-13623613231168444] Matson J. L. (Ed.). (2016). Comorbid conditions among children with autism spectrum disorders. Springer.

[bibr55-13623613231168444] Mazurek M. O. (2014). Loneliness, friendship, and well-being in adults with autism spectrum disorders. Autism, 18(3), 223–232. 10.1177/1362361312474121 24092838

[bibr56-13623613231168444] Monteiro A. P. T. D. A. V . (2011). Psychometric properties of a Russian version of the Social Support Questionnaire (SSQ6) in eastern European immigrants. Contemporary Nurse, 39(2), 157–162. 10.5172/conu.2011.157 22551428

[bibr57-13623613231168444] Nicolaidis C. Kripke C. C. Raymaker D. (2014). Primary care for adults on the autism spectrum. Medical Clinics of North America, 98(5), 1169–1191. 10.1016/j.mcna.2014.06.011 25134878 PMC4851469

[bibr58-13623613231168444] Nicolaidis C. Raymaker D. McDonald K. Dern S. Boisclair W. C. Ashkenazy E. Baggs A. (2013). Comparison of healthcare experiences in autistic and non-autistic adults: A cross-sectional online survey facilitated by an academic-community partnership. Journal General International Medicine, 28(6), 761–769. 10.1007/s11606-012-2262-7 PMC3663938 23179969

[bibr59-13623613231168444] Nicolaidis C. Raymaker D. McDonald K. Kapp S. Weiner M. Ashkenazy E. Gerrity M. Kripke C. Platt L. Baggs A. (2016). The development and evaluation of an online healthcare toolkit for autistic adults and their primary care providers. Journal of General Internal Medicine, 31(10), 1180–1189. 10.1007/s11606-016-3763-6 27271730 PMC5023610

[bibr60-13623613231168444] Nicolaidis C. Raymaker D. M. Ashkenazy E. McDonald K. E. Dern S. Baggs A. E. V. Kapp S. K. Weiner M. Boisclair W. C. (2015). ‘Respect the way I need to communicate with you’: Healthcare experiences of adults on the autism spectrum. Autism, 19(7), 824–831. 10.1177/1362361315576221 25882392 PMC4841263

[bibr61-13623613231168444] Park S. H. Demetriou E. A. Pepper K. L. Song Y. J. C. Thomas E. E. Hickie I. B. Glozier N. Guastella A. J. (2019). Validation of the 36-item and 12-item self-report World Health Organization Disability Assessment Schedule II (WHODAS-II) in individuals with autism spectrum disorder. Autism Research, 12(7), 1101–1111. 10.1002/aur.2115 31033250

[bibr62-13623613231168444] Patak L. Wilson-Stronks A. Costello J. Kleinpell R. M. Henneman E. A. Person C. Happ M. B. (2009). Improving patient-provider communication: A call to action. The Journal of Nursing Administration, 39(9), 372–376. 10.1097/NNA.0b013e3181b414ca 19745632 PMC2904301

[bibr63-13623613231168444] Pierson M. E. Prenoveau J. M. Craske M. G. Netsi E. Stein A. (2017). Psychometric properties of the Generalized Anxiety Disorder Questionnaire – IV (GAD-Q-IV) in postpartum mothers. Psychological Assessment, 29(11), 1391–1399. 10.1037/pas0000443 28221055 PMC5695386

[bibr64-13623613231168444] Rødgaard E.-M. Jensen K. Miskowiak K. W. Mottron L. (2022). Representativeness of autistic samples in studies recruiting through social media. Autism Research, 15(8), 1447–1456. 10.1002/aur.2777 35809003 PMC9541916

[bibr65-13623613231168444] Raymaker D. M. McDonald K. E. Ashkenazy E. Gerrity M. Baggs A. M. Kripke C. Hourston S. Nicolaidis C. (2017). Barriers to healthcare: Instrument development and comparison between autistic adults and adults with and without other disabilities. Autism, 21(8), 972–984. 10.1177/1362361316661261. 27663266 PMC5362353

[bibr66-13623613231168444] Rider G. N. McMorris B. J. Gower A. L. Coleman E. Brown C. Eisenberg M. E. (2019). Perspectives from nurses and physicians on training needs and comfort working with transgender and gender-diverse youth. Journal of Pediatric Health Care, 33(4), 379–385. 10.1016/j.pedhc.2018.11.003 30827755 PMC6589105

[bibr67-13623613231168444] Riggs D. W. Coleman K. Due C. (2014). Healthcare experiences of gender diverse Australians: A mixed-methods, self-report survey. BMC Public Health, 14(1), Article 230. 10.1186/1471-2458-14-230 PMC3973980 24597614

[bibr68-13623613231168444] Rothman K. J. (1990). No adjustments are needed for multiple comparisons. Epidemiology, 1(1), 43–46. 2081237

[bibr69-13623613231168444] Royal Commission into Violence, Abuse, Neglect and Exploitation of People with Disability. (2020). Interim report. https://disability.royalcommission.gov.au/publications/interim-report

[bibr70-13623613231168444] Santhanam S. P. Hewitt L. E. (2021). Perspectives of adults with autism on social communication intervention. Communication Disorders Quarterly, 42(3), 156–165. 10.1177/1525740120905501

[bibr71-13623613231168444] Serban N. (2019). Healthcare system access: Measurement, Inference, and Intervention. John Wiley & Sons.

[bibr72-13623613231168444] Sheu S. J. Wei I. L. Chen C. H. Yu S. Tang F. I. (2009). Using snowball sampling method with nurses to understand medication administration errors. Journal of Clinical Nursing, 18(4), 559–569. 10.1111/j.1365-2702.2007.02048.x 18298506

[bibr73-13623613231168444] Silveira M. F. Almeida J. C. Freire R. S. Haikal D. S. Martins A. E. de B. L. (2013). Psychometric properties of the quality of life assessment instrument: 12-item health survey (SF-12). Ciencia & Saude Coletiva, 18(7), 1923–1931. 10.1590/s1413-81232013000700007 23827896

[bibr74-13623613231168444] Sousa R. M. Dewey M. E. Acosta D. Jotheeswaran A. T. Castro-Costa E. Ferri C. P. Guerra M. Huang Y. Jacob K. S. Pichardo J. G. R. Ramírez N. G. Rodriguez J. L. Rodriguez M. C. Salas A. Sosa A. L. Williams J. Prince M. J. (2010). Measuring disability across cultures: The psychometric properties of the WHODAS II in older people from seven low- and middle-income countries – The 10/66 dementia research group population-based survey. International Journal of Methods in Psychiatric Research, 19(1), 1–17. 10.1002/mpr.299 PMC2896722 20104493

[bibr75-13623613231168444] Taylor H. Ingham B. Mason D. Finch T. Wilson C. Scarlett C. Moss S. Buckley C. Urbanowicz A. Raymaker D. Seiboth C. Lees R. Garland D. Osbourne M. Lennox N. Cooper S.-A. Nicolaidis C. Parr J. R. (2022). Co-design of an NHS primary care health check for autistic adults. Autism. 27(4). 10.1177/13623613221132921 PMC10115930 36409011

[bibr76-13623613231168444] Tobin M. C. Drager K. D. R. Richardson L. F. (2014). A systematic review of social participation for adults with autism spectrum disorders: Support, social functioning, and quality of life. Research in Autism Spectrum Disorders, 8(3), 214–229. 10.1016/j.rasd.2013.12.002

[bibr77-13623613231168444] Vohra R. Madhavan S. Sambamoorthi U. (2017). Comorbidity prevalence, healthcare utilization, and expenditures of Medicaid enrolled adults with autism spectrum disorders. Autism, 21(8), 995–1009. 10.1177/1362361316665222 27875247 PMC5517354

[bibr78-13623613231168444] Walsh C. Lydon S. Hehir A. O’Connor P. (2020). Development and evaluation of a novel caregiver-report tool to assess barriers to physical healthcare for people on the autism spectrum. Research in Autism Spectrum Disorders, 79, Article 101680. https://doi.org/https://doi.org/10.1016/j.rasd.2020.101680 10.1016/j.rasd.2020.101680 PMC7554131 33072182

[bibr79-13623613231168444] Walsh C. Lydon S. O’Dowd E. O’Connor P. (2020). Barriers to healthcare for persons with autism: A systematic review of the literature and development of a taxonomy. Developmental Neurorehabilitation, 23(7), 413–430. 10.1080/17518423.2020.1716868 36112897

[bibr80-13623613231168444] Warrier V. Greenberg D. M. Weir E. Buckingham C. Smith P. Lai M.-C. Allison C. Baron-Cohen S. (2020). Elevated rates of autism, other neurodevelopmental and psychiatric diagnoses, and autistic traits in transgender and gender-diverse individuals. Nature Communications, 11(1), Article 3959. 10.1038/s41467-020-17794-1. PMC7415151 32770077

[bibr81-13623613231168444] World Health Organization. (2008). World health report 2008: Primary health care now more than ever.

[bibr82-13623613231168444] Wu C.-H. Yao G. (2008). Psychometric analysis of the short-form UCLA Loneliness Scale (ULS-8) in Taiwanese undergraduate students. Personality and Individual Differences, 44(8), 1762–1771. 10.1016/j.paid.2008.02.003

[bibr83-13623613231168444] Zerbo O. Massolo M. L. Qian Y. Croen L. A. (2015). A study of physician knowledge and experience with autism in adults in a large integrated healthcare system. Journal of Autism and Developmental Disorders, 45(12), 4002–4014. 10.1007/s10803-015-2579-2 26334872

